# Supplementary material for: Uranium carbonate complexes demonstrate drastic decrease in stability at elevated temperatures
Source: Commun Chem. 2021 Aug 16;4:120. doi: 10.1038/s42004-021-00558-3 (PMC9814475; doi:10.1038/s42004-021-00558-3)
Supplement: Supplementary file 1 — Supplementary Information [file 42004_2021_558_MOESM1_ESM.pdf]

Supplementary Methods and Results

Raman Spectroscopy

Spectra collected at 25, 50 and 98 ºC can be seen in Supplementary Supplementary Figure 1, showing no difference in the aqueous species after extended exposure to temperature. Raw data may be found in the electronic supplement data file.

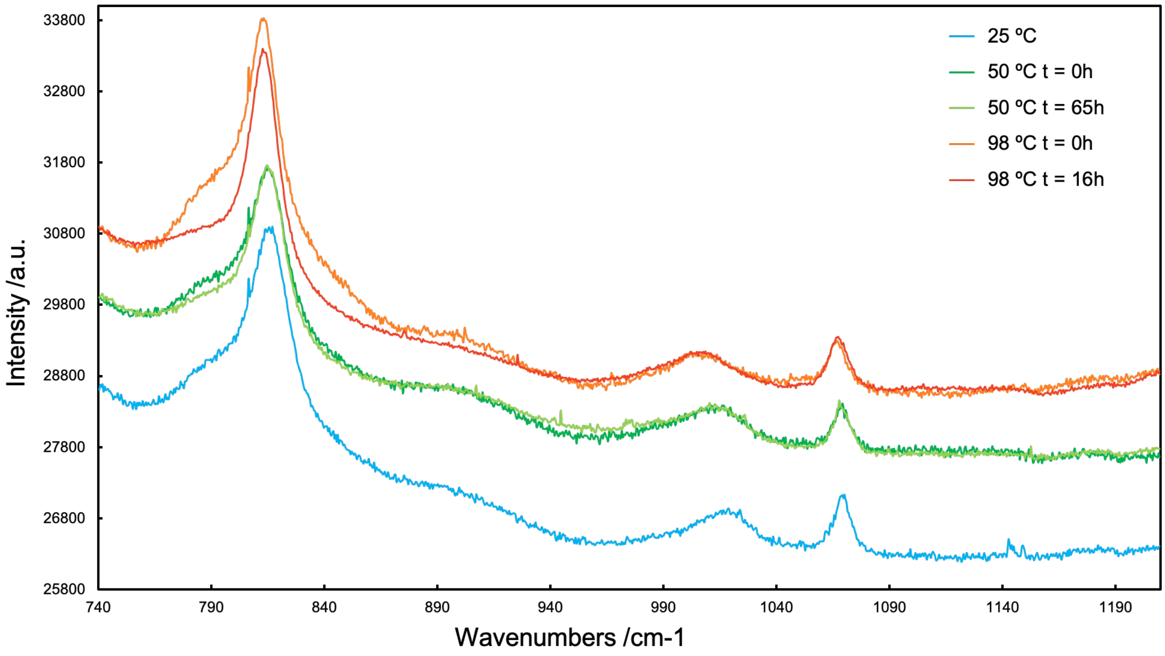

Supplementary Figure 1. Raman spectra of 0.012 m UO_2^{2+} + 0.1 m $NaHCO_3$ taken at 25, 50 and 98 ºC, before and after heating.

XAS Experiments

Full fitting of the EXAFS was not performed and instead these paths were used to obtain a qualitative understanding of the coordination chemistry of this solution. Two main peaks are observable in the FT data, which arise due to single-scattering paths corresponding to two axial oxygen atoms, a variable number of equatorial oxygen atoms (6 in the case of the uranyl tricarbonate complex at ambient pressure and temperature). The axial oxygen atoms correspond to the oxygen atoms of the UO_2^{2+} cation and the equatorial oxygen atoms are from the CO_3^{2-} and H_2O molecules that coordinate around the equator of the UO_2^{2+} ion. A third peak manifesting as a shoulder to the right of the O_{eq} peak, represents the contribution due to single scattering with the carbon atoms (i.e., U - C single scattering) associated with CO_3^{2-} ligands. As temperature increases, a clear decrease in the intensity of this shoulder is observed, indicating a potential reduction in CO_3^{2-} complexation at elevated temperatures. By 125 °C, the intensity of this feature was minimal. Also of note is a general decrease in the intensity of the FT spectra with increasing temperatures potentially linked to changing complexation of the system. This general trend of decreased prevalence of uranyl carbonate at elevated temperatures agrees qualitatively with solubility measurements reported in this work.

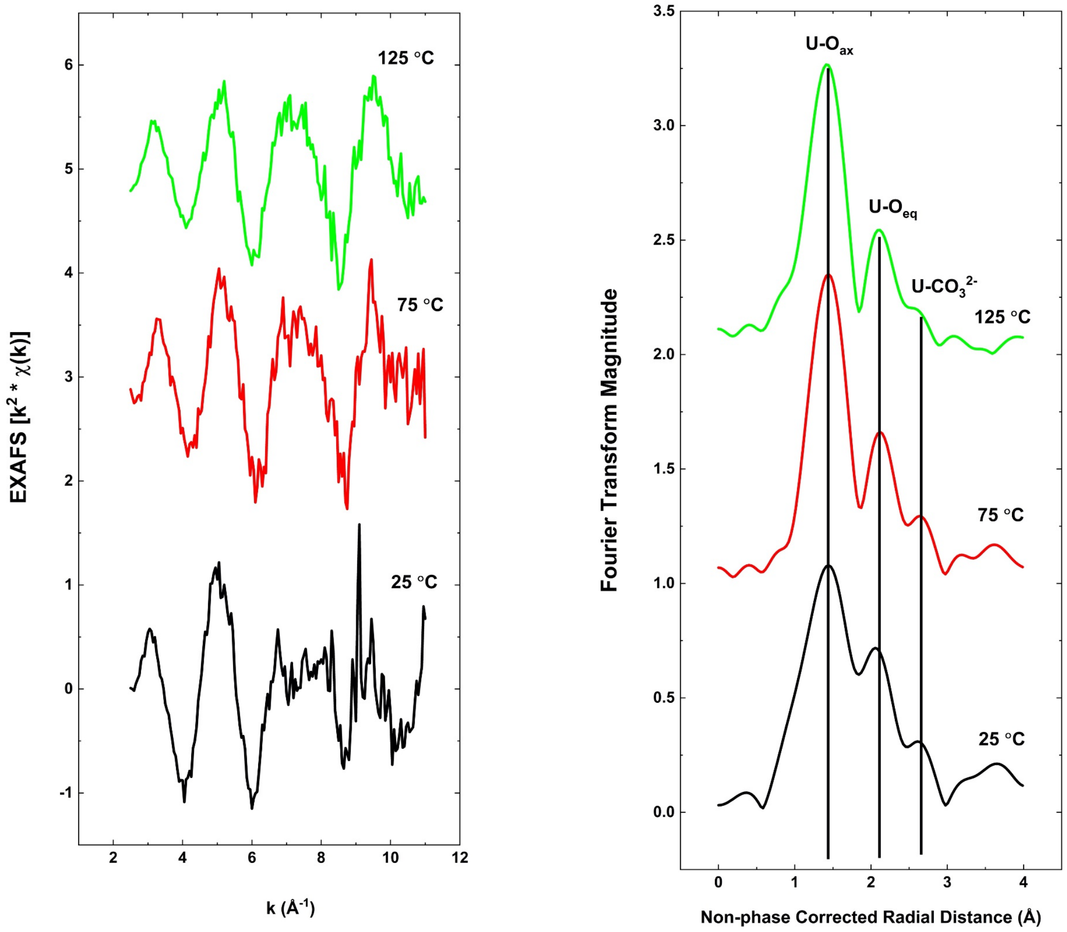

(a) **(b)**

Supplementary Figure 2. EXAFS spectra and corresponding Fourier transforms.

(a) *U L_{III}-edge k^2-weighted EXAFS spectra (k = 3.0 - 10.5 Å^{-1}) from 25 °C to 125 °C* ***(b)*** *Corresponding Fourier transform of EXAFS spectra. Three main features are observable corresponding to single-scattering paths with respect to the uranium absorber atom and two axial oxygen (O_{ax}) atoms, a variable number of equatorial oxygen (O_{eq}) atoms, and a variable number of carbon atoms (U-CO_3^{2-}). With increasing temperature, the intensity corresponding to the U-CO_3^{2-} scattering path decreases indicating a loss of carbonate complexation.*

<u>*Solubility Experiments – Further methodological details*</u>

Compositions of solutions from both series are reported in Supplementary Supplementary Table 1 along with recorded uranium solubilities, calculated pHs at the experimental temperature and pH corrected uranium concentrations. A schematic diagram of the autoclave apparatus used in our solubility experiments is presented in Supplementary Supplementary Figure 3 and a diagram illustrating the key steps of the method is presented in Supplementary

Supplementary Figure 4. To ensure the measured concentrations were truly indicative of a state of equilibrium between the reference solid and experimental solution a series of kinetic experiments were conducted at 200 ºC. This temperature was chosen at it represented the lowest temperature investigated using the solubility technique; it was assumed that equilibrium would be achieved over a shorter time period at higher temperatures. A series of autoclaves each containing identical concentrations of $NaHCO_3$ (0.2 *m*) were heated to 200 ºC after the initial 150 ºC ramp period. Individual autoclaves were then periodically extracted and quenched at daily intervals permitting the evaluation of the change in dissolved uranium as a function of time. Measured uranium concentrations from these kinetics

experiments are reported in Supplementary Figure 5 and suggest that equilibrium between the reference solid and experimental solutions was achieved within 1 day - this allowed us to assert that results derived from autoclaves heated for 5 days were truly representative of the equilibrium state between reference solid and solution.

Supplementary Table 1. Compositions and results from all autoclave solubility solutions. All concentrations are reported as mol/kg of H_2O. pH Values were calculated for the experimental temperature using the modified extended Debye Hückel model - system definition and thermodynamic molal property details are discussed further below. pH corrected values were normalised to a pH of 8 and were made based on a 1:1 dependence of log uranium concentration on pH as suggested by results reported in Figure 4 in the main text.

$\underline{T}$ (ºC)	[NaCl]	[$NaHCO_3$]	[Na_2CO_3]	log m(U)	pH_T	Log m(U) (pH norm)
200	1	0.02	0	-5.09	7.81	-5.28
200	1	0.01	0	-5.10	7.69	-5.41
200	1	0.02	0	-5.95	7.81	-6.14
200	1	0.05	0	-5.95	7.95	-6.01
200	1	0.1	0	-5.84	8.01	-5.82
200	1	0.200	0	-5.44	8.05	-5.39
200	1	0.010	0	-4.56	7.69	-4.87
200	1	0.010	0	-4.96	7.69	-5.27
200	1	0.100	0	-5.24	8.01	-5.22
200	1	0.100	0	-5.33	8.01	-5.32
200	1	0.1	0	-4.58	8.01	-4.56
200	2	0.05	0	-5.44	7.84	-5.60
200	2	0.1	0	-4.80	7.92	-4.88
200	2	0.15	0	-4.59	7.95	-4.64
200	2	0.3	0	-4.51	7.98	-4.52
200	1	0.2	0	-4.83	8.06	-4.77
200	1	0.2	0	-5.13	8.06	-5.06
200	1	0.2	0	-5.19	8.06	-5.13
200	1	0.2	0	-5.27	8.06	-5.21
200	1	0.2	0	-5.05	8.06	-4.99
200	1	0.2	0	-4.76	8.06	-4.70
200	1	0.2	0	-5.55	8.06	-5.49
200	2	0	0.3	-6.98	9.78	-5.19
200	2	0.1	0.2	-7.58	9.51	-6.06
200	2	0.1	0.2	-8.21	9.51	-6.70
200	2	0.15	0.15	-7.69	9.34	-6.35
200	2	0.15	0.15	-7.490	9.34	-6.15
200	2	0.2	0.1	-6.74	9.12	-5.62
200	2	0	0.1	-6.82	9.49	-5.33
200	2	0	0.2	-6.95	9.68	-5.28
200	2	0	0.3	-8.39	9.78	-6.61
200	2	0	0.4	-7.13	9.85	-5.28
200	2	0	0.5	-6.53	9.91	-4.63
250	1	0.001	0	-4.69	7.22	-5.47
250	1	0.005	0	-4.79	7.63	-5.16
250	1	0.01	0	-6.34	7.79	-6.55
250	1	0.03	0	-5.67	8.02	-5.65
250	1	0.05	0	-5.94	8.12	-5.82
250	1	0.08	0	-6.68	8.20	-6.48
250	1	0.1	0	-6.08	8.23	-5.85
250	1	0.001	0	-6.15	7.22	-6.93
250	1	0.005	0	-6.94	7.63	-7.32
250	1	0.01	0	-6.95	7.79	-7.17
250	1	0.1	0	-7.17	8.23	-6.94
250	1	0.2	0	-5.72	8.32	-5.40
250	1	0.3	0	-5.81	8.35	-5.46
250	1	0.4	0	-6.94	8.37	-6.57
250	2	0.2	0	-6.76	8.20	-6.56

250	2	0.4	0	-7.36	8.27	-7.09
250	2	0.5	0	-6.87	8.28	-6.59
250	2	0.6	0	-7.42	8.29	-7.13
250	2	0.65	0	-7.67	8.30	-7.37
250	2	0.7	0	-7.04	8.30	-6.74
250	2	0.75	0	-7.42	8.30	-7.18
250	2	0.8	0	-6.55	8.30	-6.25
250	2	0.1	0.2	-7.39	9.52	-5.87
250	2	0.1	0.2	-7.32	9.52	-5.80
250	2	0.15	0.15	-6.87	9.38	-5.49
250	2	0.2	0.1	-7.34	9.20	-6.15
250	2	0.2	0.1	-7.57	9.20	-6.37
250	2	0.3	0	-6.39	8.25	-6.15

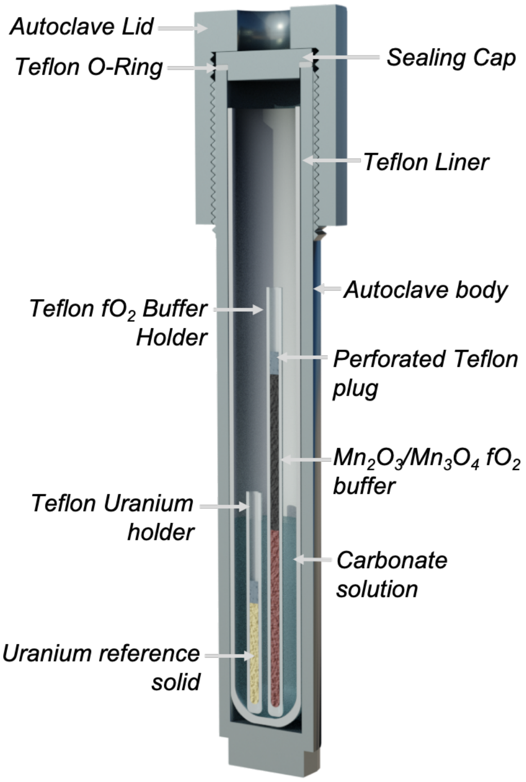

Supplementary Figure 3. Schematic diagram of the autoclave system used for solubility experiments.

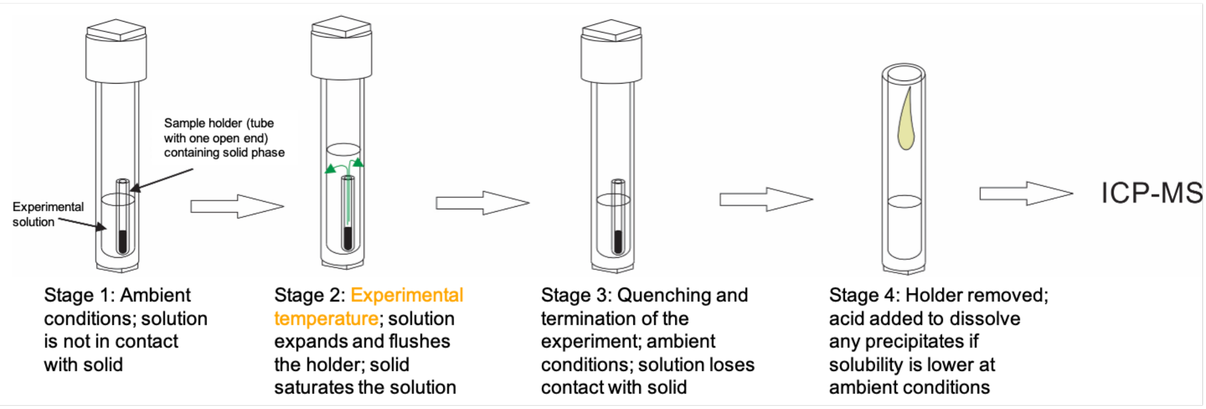

Supplementary Figure 4. Major steps in the autoclave solubility technique.

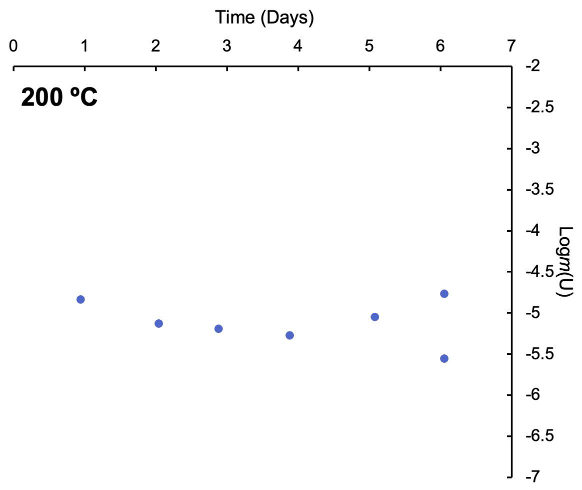

Supplementary Figure 5. *Results from the kinetic series conducted at 200 °C. Note that equilibrium was essentially reached within 1 day.*

Data Processing Procedure - Derivation of formation constants

Based on the gradients (-1.2 and -0.77 at 200 °C and 250 °C, respectively) of the measured pH-solubility relationships reported in Figure 4 in the main text, we suggest the predominant species controlling uranium solubility at the experimental conditions to be UO_2OH^+ forming via the reaction described in Supplementary Equation 1.

$$UO_2(OH)_{2(cr)} + H^+ \rightleftharpoons UO_2OH^+ + H_2O \qquad (1)$$

To derive the Gibbs free energy of formation of UO_2OH^+ at both 200 and 250 °C the OptimA program (part of the HCh software package[1,2]) was utilised. This required a chemical system definition and activity model, both of which we have described in the main text. Through a least-squares optimisation procedure (i.e. minimising deviation between experimental and measured values of uranium solubility by varying the Gibbs free energy of the UO_2OH^+ complex) OptimA was used to derive the Gibbs free energies of formation of UO_2OH^+ as well as the uncertainty of this parameter at both 200 and 250 °C . These Gibbs free energies were then used to calculate formation constants ($\log\beta_1$) for Supplementary Equation 2 at those temperatures. Derived formation constants are reported in Supplementary Table 2 alongside formation constants for the same reaction reported by Zanonato et al. (2004)[3].

$$UO_2^{2+} + OH^- \rightleftharpoons UO_2OH^+ \quad \log\beta_1 = \log a_{UO_2OH^+} - \log a_{OH^-} - \log a_{UO_2^{2+}} \qquad (2)$$

Supplementary Table 2. Formation constants for Supplementary Equation 2. [a]Values reported by Zanonato et al. (2004)[3]. [b]Values derived in this work.

Temperature (°C)	25[a]	40[a]	55[a]	70[a]	85[a]	200[b]	250[b]
$\log\beta_1$	8.60	8.62	8.27	8.50	8.49	11.81	11.33
Uncertainty	±0.24	±0.11	±0.24	±0.11	±0.15	±0.52	±0.57

To permit the interpolation and limited extrapolation of the formation constants for Supplementary Equation 2, all values reported in Supplementary Table 2 have been fitted to the modified Ryzhenko-Bryzgalin[2,4] (MRB) model (Supplementary Equation 3) using the OptimC program (also a part of the

HCh software package). K is the dissociation constant of the ion pair and T_r and P_r refer to the reference temperature and pressure conditions. $B_{(T,P)}$ accounts for changes in the properties of water with pressure and temperature and is calculated using values from Marshall and Franck (1981)[5]. $A_{zz/a}$ and $B_{zz/a}$ are both empirical parameters derived by fitting experimental data to the model. Derived MRB parameters are reported in Supplementary Table 3. Using these newly derived parameters we have tabulated new formation constants for Supplementary Equation 2 from 25 to 250 ºC, these are reported in Supplementary Table 4.

$$\log K_{(T,P)} = \frac{T_r}{T} \log K_{(T_r,P_r)} + B_{(T,P)} * \left(A_{zz/a} + \frac{B_{zz/a}}{T} \right) \qquad (3)$$

Supplementary Table 2. MRB fitting parameters for the UO_2OH^+ dissociation reaction using values reported in Supplementary Table 2.

Species	pK(298)	A(zz/a)	B(zz/a)
UO_2OH^+	8.334	2.279	-7.47

Supplementary Table 3. Formation constants of Supplementary Equation 2 at a range of temperatures calculated using MRB parameters reported in Supplementary Table 3.

T (ºC)	$\log\beta_1$
25	8.33
50	8.50
75	8.76
100	9.08
125	9.44
150	9.85
175	10.29
200	10.78
225	11.33
250	11.96

Supplementary References

1 Shvarov, Y. A suite of programs, OptimA, OptimB, OptimC, and OptimS compatible with the Unitherm database, for deriving the thermodynamic properties of aqueous species from solubility, potentiometry and spectroscopy measurements. *Appl. Geochem.* **55**, 17-27 (2015).

2 HCh, A Software Package for Geochemical Equilibrium Modeling: User's Guide. Record 1999/25 (1999).

3 Zanonato, P. *et al.* Hydrolysis of Uranium(VI) at Variable Temperatures (10-85 °C). *Journal of the American Chemical Society* **126**, 5515-5522 (2004).

4 Ryzhenko, B. N., Bryzgalin, O. V., Artamkina, I. Y., Spasennykh, M. & Shapkin, A. I. An electrostatic model for the electrolytic dissociation of inorganic substances dissolved in water. *Geochem. Int.* **22**, 138-144 (1985).

5 Marshall, W. L. & Franck, E. U. Ion product of water substance, 0–1000 °C, 1–10,000 bars New International Formulation and its background. *J. Phys. Chem. Ref. Data* **10**, 295-304 (1981).
