# Supplementary material for: Uranium carbonate complexes demonstrate drastic decrease in stability at elevated temperatures
Source: Commun Chem. 2021 Aug 16;4:120. doi: 10.1038/s42004-021-00558-3 (PMC9814475; doi:10.1038/s42004-021-00558-3)
Supplement: Supplementary file 4 — Description of Additional Supplementary Files [file 42004_2021_558_MOESM4_ESM.pdf]

Description of Additional Supplementary Files

File name: Supplementary Data 1

Description: An Excel file containing consolidated data obtained in this study. The data are subdivided on tabs for Solubility data, Calculated solution compositions, Raman data, XAS data, and Solubility phase XRD data.
